# User-Oriented ICT Cloud Architecture for High-Accuracy GNSS-Based Services

**DOI:** 10.3390/s19112635

**Published:** 2019-06-11

**Authors:** Hossein Ghobadi, Paola Testa, Luca Spogli, Massimo Cafaro, Lucilla Alfonsi, Vincenzo Romano, Richard Bru

**Affiliations:** 1 Istituto Nazionale di Geofisica e Vulcanologia, 00143 Rome, Italy; luca.spogli@ingv.it (L.S.); lucilla.alfonsi@ingv.it (L.A.); vincenzo.romano@ingv.it (V.R.); 2 Department of Engineering for Innovation, University of Salento (UNISALENTO), 73100 Lecce, Italy; massimo.cafaro@unisalento.it; 3 Department of Strategy, Toulouse Business School (TBS), 31068 Toulouse, France; paola.testa@noveltis.fr; 4 Noveltis, 31670 Toulouse, France; richard.bru@noveltis.fr; 5 SpacEarth Technology (SET), 00143 Rome, Italy

**Keywords:** ICT, GNSS, error mitigation, cloud-based service, integrity, scalability, market trends, servitization

## Abstract

We introduce a new information and communication technology (ICT) cloud-based architecture for Global Navigation Satellite System (GNSS) high-accuracy solutions, offering also a commercial overview of GNSS downstream market to show how the developed innovation is thought to fit in the real context. The designed architecture is featured by dynamic scalability, increased integrity, and greater agility of the ICT system. The novelty of the solution developed is a customized ICT architecture, obtained through unique and privileged access to user communities in the frame of the H2020 project TREASURE, allowing the development of a solution entirely driven by user needs. The economic outlook of GNSS downstream markets evolution highlights how the technology proposed effectively matches the evolving business environment, specifically in regard to the increasing need for flexibility and competitive advantage deriving from services. The simultaneous adoption of the technical and commercial perspective is meant to offer interesting findings to both the scientific community and GNSS industry, creating synergies previously unexplored.

## 1. Introduction

Global Navigation Satellite System (GNSS) technology [1] has a growing prominent role in the international horizon, both in terms of technical development and regarding new domains of application [2]. This ever- and fast-evolving scenario brings along new opportunities and challenges for researchers and commercial players. In particular, the concomitant necessity to address user needs (soft sciences) and consistently invest in technical innovation (hard sciences) cannot be neglected. 

Adopting these two complementary perspectives, the present article proposes a new information and communication technology (ICT) cloud-based architecture for GNSS high-accuracy solutions, offering also a commercial overview to show how the developed innovation fits in the present context. Therefore, this work has implications for both researchers and businessmen.

To this end, a novel and unique methodology is adopted: The authors’ participation in the H2020 project termed “Training Research and Applications Network to Support the Ultimate Real-Time High-Accuracy EGNSS Solution” (TREASURE. http://www.treasure-gnss.eu accessed on 31 May 2019) [3] offers a privileged access to a wide GNSS user community involving big corporates, small and medium enterprises (SMEs), start-ups, universities and research centers. In particular, TREASURE will advance the state of the art in European GNSS, by improving the accuracy of GNSS positioning techniques, providing different GNSS high-accuracy products on atmospheric (ionospheric and tropospheric) corrections, orbits and clocks so that they can be introduced as part of a commercial service in the future. These products will be realized by early-stage researchers (ESRs), who are at the very center of the TREASURE training network, each of them offering a research contribution to the common goal of GNSS high-precision technology advancement, in terms of scientific, technical and commercial perspectives. To fulfill their objectives, ESRs need an ICT infrastructure to provide potential adopters with their improved algorithms. 

Relying on this network, relevant user (i.e., the ESRs) needs have been identified, to drive the design of an ICT cloud infrastructure for GNSS-based solutions, characterized by scalability, flexibility and reliability [4] of the ICT system with respect to the state of the art. Therefore, the present work investigates a suitable and interoperable ICT infrastructure for the exploitation of TREASURE’s innovative algorithms and its ultimate real-time high-accuracy EGNSS (European GNSS–Galileo) solution.

The technological proposition is complemented by an investigation of GNSS downstream markets trends and practical considerations to develop a suitable go-to-market strategy. 

Existing services supporting high-accuracy GNSS-based solutions are very few and they are typically designed to provide data and/or products that users, mainly service providers, can then include in their own infrastructure/service. This is the case of the well-known and widely used IGS products (http://www.igs.org/products accessed on 31 May 2019) [5], which include GNSS observables, satellite orbits and clocks, broadcast ephemeris files, Earth’s rotation and atmospheric parameters (tropospheric zenith path delay and ionospheric total electron content). Despite the wide range of applications and experimentation covered by IGS, the provided products cannot be customized and are not designed for specific GNSS users. 

Recently, in the frame of the Ionosphere Prediction Service (IPS) project [6] a prototype service (available at https://ips.telespazio.com accessed on 31 May 2019) [7] has been designed and implemented with the aim of translating the nowcasting or forecasting information of the space weather conditions into products fine-tuned for user communities, aviation in particular. Specifically, in IPS the performance of the Satellite-Based Augmentation System (SBAS) and Aircraft-Based Augmentation System (ABAS) are nowcasted (both) and forecasted (ABAS only). It provides the users with the real-time performance of the European Geostationary Navigation Overlay Service (EGNOS) service with a graphical representation.

According to the authors’ best knowledge, except for the IPS system, exclusively dedicated to the aviation community, there is no currently available similar system to the one proposed in the present study, which aims at tuning the design of an ICT infrastructure for GNSS-based solution according to surveyed and identified user needs. Therefore, the necessity of introducing a new ICT infrastructure is stressed out in the current work.

Albeit today’s GNSS ICT infrastructures are mainly on-premises, recent surveys performed by the authors with the support of TREASURE partners and ESRs, show that the industry is moving towards virtual machines and cloud-based ICT solutions to achieve a scalable and reliable ICT architecture. Cloud services are typically categorized in platform as a service (PaaS), software as a service (SaaS) and infrastructure as a service (IaaS) for public, private, and hybrid cloud architectures. Being design-free, cloud technology is also easy to customize. The growing demand for seamless, ubiquitous, and reliable GNSS positioning services calls for improved ICT technology [8]. Bearing this in mind, here the goal is to introduce the design of a global, cutting-edge, and service-oriented platform for GNSS error corrections diffusion.

ICT requirements of GNSS users are continuously evolving, especially in terms of functionalities and compatibility with complementary and new emerging technologies. These needs can be addressed in a cloud-based platform by updating the cloud software, regardless of the user terminals [9]. The adopted approach is compatible with both real-time and post-processing methods. We propose virtual containers where innovative GNSS error mitigation algorithms are deployed as microservices to deliver high-accuracy positioning as final product, ensuring the integrity of the system, since the failure of a single application program interface (API) does not affect the whole system performance.

The ICT architecture comprises data acquisition, algorithms for product generation, preprocessing and data consolidation, product distribution for real-time processing and product archiving for post-processing. It is, therefore, a comprehensive and versatile solution, which can support the most important techniques currently used for GNSS high precision positioning (precise point positioning (PPP), real-time kinematic (RTK), PPP-RTK) [10,11], on a local, regional or global scale, according to the customer requirements. The necessity of a flexible ICT infrastructure has been highlighted, among others, by [12], as support to heterogeneous satellite-based services, which might satisfy several purposes in different applications segments such as e-health, smart mobility, and emergency management.

Being inspired by the work in [13], in which only a research-driven application has been implemented on cloud infrastructure, seven use cases from different GNSS domains (from research to industry) have been considered and integrated among them. In addition, while the proposed solution in [13] was the use of a private cloud on a pool of virtual machines running on the servers at a research institute, here we propose a solution based on a public type of cloud. The design-free option promoted by public cloud makes the deployment of the microservices easier and cost-effective system maintenance in comparison to the private cloud, and this is because security and network design are pre-defined in a public cloud.

We notice readers that the realization of such a system and the evaluation of its performance are out of the scope of this paper. This somehow limits the possibility also to test the whole system performance in terms of timing, introduced delays, and jitters and then provide a thorough comparison with the benchmarks reported in [13]. However, system components and required performance are assessed through a technical survey submitted to TREASURE’s partners and ESRs.

An added value of this work is the complementary and multi-disciplinary analysis of the commercial potential of the innovation proposed. As stated in [13] in relation to the proposed Ground-Based Scintillation Climatology (GBSC) software tool, the emergence of new user needs implies also business opportunities. Namely, malleability and optimized computational capacity of a cloud-based ICT infrastructure can pave the way for the provision of better-performing and affordable services in new domains of application. The performance of a market assessment highlights the business potential of the proposed ICT cloud infrastructure, on top of its technical appeal. 

The paper is structured as follows: Section 2 introduces materials and methods, in which ICT technical feasibility developed to study users’ needs and the mitigation algorithms are described. Section 3 deals with the results, in particular concerning the ICT architecture design and its commercial perspectives. In Section 4 the achieved results are discussed, followed by the conclusions in Section 5.

## 2. Materials and Methods

**Figure 1:**
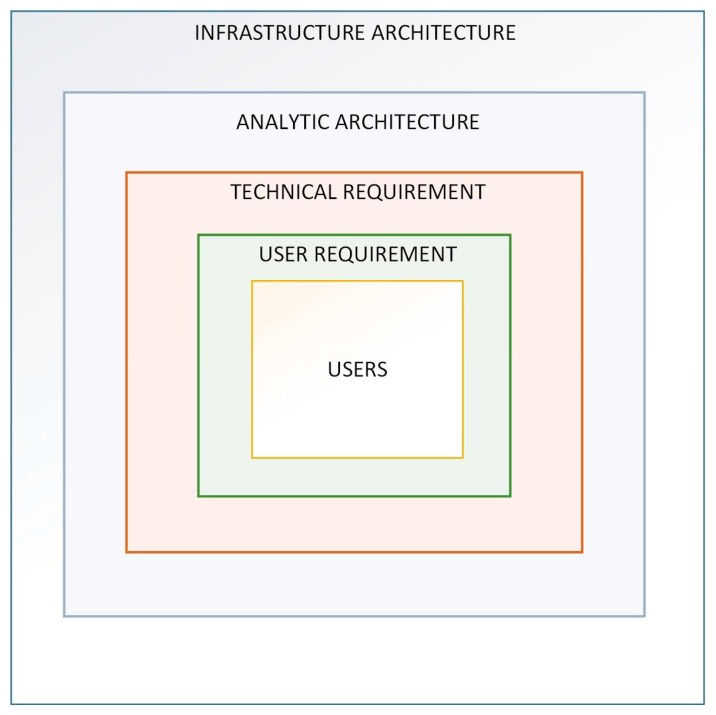
Technical feasibility approach.

For the feasibility study and the design of the cloud-based ICT infrastructure, a hierarchical approach has been adopted, as shown in Figure 1. It shows that to develop an ICT architecture, firstly users and their needs must be targeted. 

The users’ needs being the core of the design, the present study took advantage of the TREASURE network by identifying a group of users among the TREASURE ESRSs. 

Each ESR (user), through his/her algorithm, provides different GNSS high-accuracy products on atmospheric (ionospheric and tropospheric), orbits, and clocks corrections. Seven algorithms were selected among the users, as reported in Table 1. 

Such algorithms, user requirements, and consequently, technical requirements were surveyed among ESRs through a questionnaire. It was composed of three sections: General information, technical information, marketing information. The technical section, which is the main focus of the questionnaire, investigated:

1. Input data—repository, communication protocols, format, size, latency, and sample rate.
2. Computation requirements—CPU, memory, programming language and compiler, execution time, storage and operating system.
3. Description of algorithm—explanation, improvement and limitations.
4. Output data—product type, format and size, repository, communication protocols, latency, and sample rate.
5. Cross-works (if any) between ESRs.

Based on the technical requirements provided by the users, an analytic architecture and infrastructure were designed and they are here presented in terms of a technical feasibility study. 

The feasibility study comprises analytical architecture and infrastructure architecture. The analytical architecture concerns the data, specifically regarding the new algorithms use and integration. The infrastructure architecture is the main focus of the paper, in order to address the emerged users’ needs with a seamless ICT architecture.

Summarizing, relying on the TREASURE network, relevant user needs were identified, which drove the development of the new ICT cloud infrastructure for GNSS-based solutions, characterized by dynamic scalability, increased integrity and greater agility of the ICT system with respect to the state of the art. According to our knowledge, there is not enough research on the infrastructures for GNSS-based solutions to do some benchmarking, reinforcing the novelty of the presented work. 

In addition, a market analysis of GNSS downstream markets, relying on secondary sources (business and academic investigations) and direct interaction with the TREASURE project partners and other stakeholders within their network (face to face interviews), allowed for the identification of important trends affecting innovation diffusion. This complementary and multi-disciplinary analysis is meant to highlight the commercial potential of the innovation proposed, on top of its technical innovativeness.

## 3. Results

### 3.1. A New ICT Infrastructure for GNSS-based Solutions

The ICT infrastructure for user-oriented GNSS high-accuracy solutions is here presented in terms of its architectural design. 

#### 3.1.1. Layout

**Figure 2:**
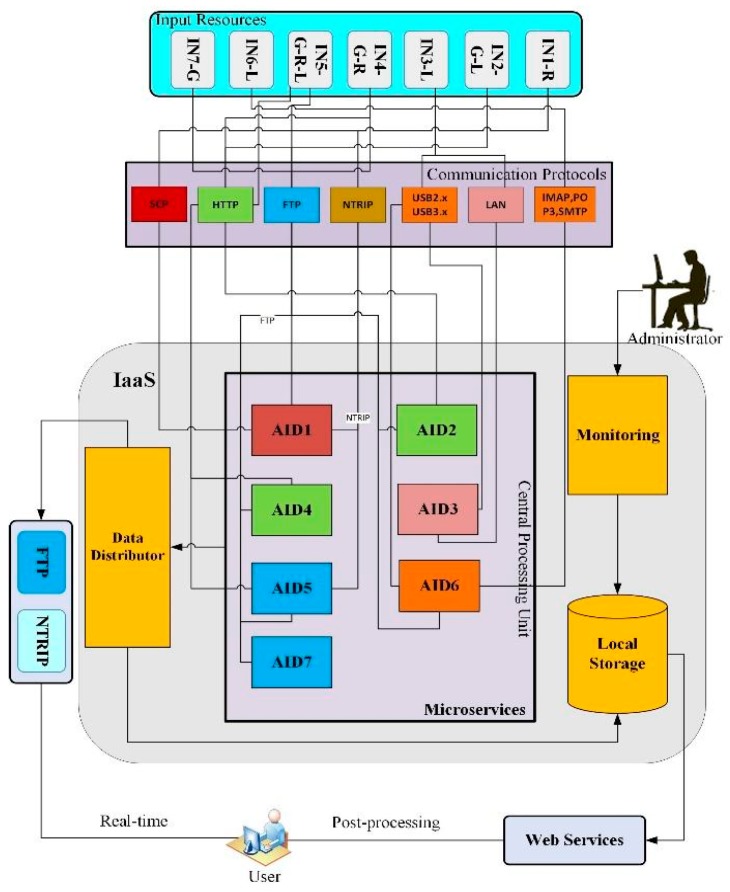
Block diagram of the designed information and communication technology (ICT) technical architecture.

The ICT system layout is designed by strictly following the processing requirements of the seven algorithms (Table 1) surveyed through a technical questionnaire, as above mentioned. The block diagram of the designed ICT technical architecture is shown in Figure 2. 

The ICT technical architecture model reveals the structure and interaction of microservices (i.e., the algorithms), and infrastructure components. The model is comprised of several architecture building blocks: Data acquisition (input resources), storage and distribution, and processing unit which are discussed in the Section 3.1.2, Section 3.1.3 and Section 3.1.4 Since we are interested in introducing a new scalable and reliable ICT infrastructure solution instead of on-premise physical infrastructure, a cloud IaaS-based service is adopted for our design. The design and operation of the ICT infrastructure are handled by an IaaS cloud provider [9]. According to the technical requirements surveyed through a questionnaire, the users need a minimum of CPU corei7 2.7 GHz, 8 GB memory and operating system-free, while most of the algorithms are MATLAB compiled software working on Windows OS. As cloud technology is elastic to users’ needs, the hardware and software requirements can be customized on the request of users. Since IaaS-based cloud services provide scalable and elastic ICT infrastructures, the hardware capabilities can be tailored to fit user needs.

#### 3.1.2. Data Acquisition, Storage and Distribution

Input data are shown in Table 2. In particular, the identifier (INPUT ID) shows the input number and one or more letters indicating the coverage of the considered networks: Global (G), regional (R), and local (L). While some data are available directly from a local network, other data are received from regional and global networks through different communication protocols. Data size is also reported in terms of the size of a single file.

In Table 2, RTCM 3.X files are standard introduced by the Radio Technical Commission for Maritime Services (RTCM. http://www.rtcm.org accessed on 31 May 2019) used internationally. RINEX (receiver independent exchange) format [14] is a standard for GNSS navigational and observational data are ASCII files. IF (intermediate frequency) data are in form of raw bits. Binary files for CIGALA/CALIBRA are in the SBF (Septentrio binary files) format, being the proprietary format of the GNSS receivers manufactured by Septentrio SSN Company. SSR (state space representation) contains time and spatial information on the GNSS state and are proprietary binary files used in the software developed by Geo++ Company to provide GNSS corrections. SP3 (standard product 3) is in ASCII format and contains the precise orbit determination of the GNSS satellites. CLK (CLoKs) is a standardized ASCII file containing clock corrections and ANTEX (antenna exchange format) is a standardized ASCII file containing satellite antenna corrections.

Input data can be obtained either from external databases, or directly from local GNSS receivers or local storages. Each microservice receives different types of data, executing its specific algorithm on them. There is a need for GNSS data manipulation to be re-evaluated and re-developed in an optimized ICT model. Hence, a new GNSS data format is needed to transmit processed data to high-accuracy users. In this regard, a new standard is proposed in [15]. 

#### 3.1.3. Processing Unit

**Figure 3:**
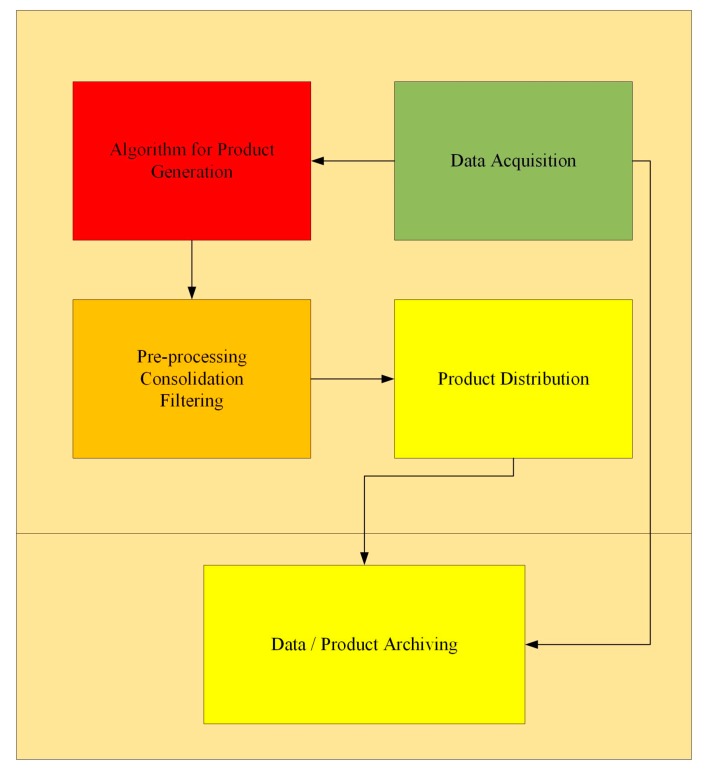
Microservice components.

To avoid system failure and the consequent need for disaster recovery, the central processing unit, not only guarantees the functioning of the whole system but also ensures the correct performance of each individual task of the microservices. Figure 3 shows the block diagram of the main components constituting each microservice. 

Microservices are composed of data acquisition, an algorithm for product generation, filtering, distribution and finally data assimilation. The functionality of a microservice is designed to fulfill the following benchmarks: Agility, dynamic scalability, and resilience. In our work, we propose the deployment of microservices on a cloud where each microservice has a task to perform, identified according to the emerging user needs collected within TREASURE. The backend of the microservices includes a container and a database. The front-end is an API gateway, to which the microservices are connected through a bus, where all microservices bring a product to the end users. 

#### 3.1.4. Data Distribution

A data distribution service provides data to users via dedicated protocols. Concerning real-time output transmission, networked transport of RTCM via internet protocol (NTRIP) and file transfer protocol (FTP) communication protocols are leveraged to meet redundancy requirements. The real-time output is accessible for GNSS high-accuracy users through a web portal for post-processing. The output data is generated in different formats, which require data integration. This can be done through an API gateway. This data is available in local storage in proportion to data sum of individuals’ microservices, monitored, and a database management model is given by an administrator. Table 3 shows the output of the proposed ICT system, in terms of the different product types: “Final”, i.e., no further computation is needed by the end-users or “intermediate”, i.e., additional processing is needed. Data types and sizes (per single file) are shown in Table 3 as well, apart from the data format already specified in Table 2, *.mat are MATLAB proprietary files.

#### 3.1.5. Security

Despite the increased complexity, securing a cloud-based microservices architecture is, by now, a highly studied problem, for which well-known solutions and common practices are available. The security objectives in the context of our ICT architecture are: Entity authentication, confidentiality, data integrity, and access control. Entity authentication refers to the process leading to the correct identification of the identity of a user. Confidentiality refers to the need to keep the information secret from all but those who are authorized to see it. Data integrity ensures that information has not been altered by unauthorized or unknown means. Access control restricts access to the resources to privileged, authorized entities. Different levels of authorization may need to be enforced, depending on the specific role of a user (role-based access control (RBAC)) [16]. 

In our ICT architecture, all of the network communications can be made secure by using different means. For instance, one can adopt Transport Layer Security (TLS) [17] or WS-Security (https://www.oasis-open.org/standards#wssv1.1.1, accessed on 31 May 2019) [18]. Regarding authentication, X.509 v3 digital certificates or Kerberos tickets may be used, along with traditional user’s ID and password credentials, may be in connection with the use of a one-time password token. The microservices can perform the authentication themselves or, alternatively, they may delegate authentication to a trusted component. The secure production identity framework for everyone (SPIFFE) is a novel attempt to simplify microservice authentication and secure network configuration. Authorization decisions may be based on several factors, e.g., the combination of the identity, some attributes, and the specific request context. In a cloud-based microservice architecture, even a single user’s request may require multiple authorization steps as it is passed from microservice to microservice. JSON web token (JWT) [19] is a simple, JSON-based (JavaScript object notation) packaging format, whose purpose is to make simple exchanging “claims”. Since the claims can represent anything legal in JSON, they can be used for access control decisions.

A solution for differentiating the security needs of the involved microservices is based on the use of API gateways (https://prometheus.io, https://aws.amazon.com/api-gateway, accessed on 31 May 2019) [20,21], which act as a front-end hiding the underlying cloud-based microservices. The basic idea is to place the gateway behind the firewall, and to place the firewall around the microservices.

Finally, the use of advanced machine learning techniques, such as those related to anomaly detection, allows monitoring and checking if the security policies are actually enforced or not, not just at the microservice level, but considering the whole distributed architecture. Anomaly detection may also automatically trigger alarms, informing system administrators or reacting autonomously to a detected attack. 

### 3.2. Fundamental Market Trends

The following paragraphs present a shortlist of the most influential dynamics that are shaping GNSS technology evolution and firms’ competitive advantage. This section is meant to offer an overview of the most impacting market evolution, also showing how the elaborated ICT cloud architecture fits in this context.

#### 3.2.1. Commoditization

Technological industries are facing the challenge of commoditization: Growing homogeneity among the offers (hardware) supplied [22]. Commoditization is an important expanding phenomenon affecting competitive leverages and potential sources of advantages [23,24]. This trend derives from more and better-informed customers and increased transparency in competitive markets, which leave more room for relatively rapid imitation, increasing the chance to cheaply switch to a different supplier. As a consequence of this osmotic process, the competitive focus partially shifts away from the core business, since the active players are forced to consider different features to remain successful.

Reference [25] showed that firms operating in commoditized sectors should heavily leverage customer intimacy, intended as a deep understanding of the customer needs and tailor the offering accordingly. This is the case of the illustrated ICT architecture for GNSS, considering the possibility it offers to tailor-make the service provided according to specific user requirements.

#### 3.2.2. Democratization

Democratization is a process through which technology becomes progressively more accessible to a growing number of people, up to the point in which users participate in the development process. As a result, products and services are more affordable and user-friendly, also thanks to mass production and digitalization. 

This phenomenon has been practically translated for GNSS downstream industry in receivers’ price decrease and improved accuracy, making mapping and navigation activities more affordable. This trend is leading the market toward the provision of integrated, highly performing, and user-friendly solutions that can fit in many different domains of applications, also thanks to modularization [2]. The proposed ICT architecture is meant to serve this purpose, thanks to its flexibility and scalability in the provision of error mitigation.

#### 3.2.3. Servitization

Manufacturers of durable goods, such as receivers or any sort of GNSS platform, increasingly try to enrich and renew their offering by complementing products with services. This trend, known as ‘servitization’ [26], refers to the tendency of manufacturing firms to “offer fuller market packages or bundles of customer-focused combinations of goods, services, support, self-service, and knowledge”. Moreover, servitization [26] and open service innovation [27] enhance the development of an organization’s innovation capabilities through the shift from products to customer-centered product-service systems, better meeting user needs and escaping the commoditization trap. 

**Figure 4:**
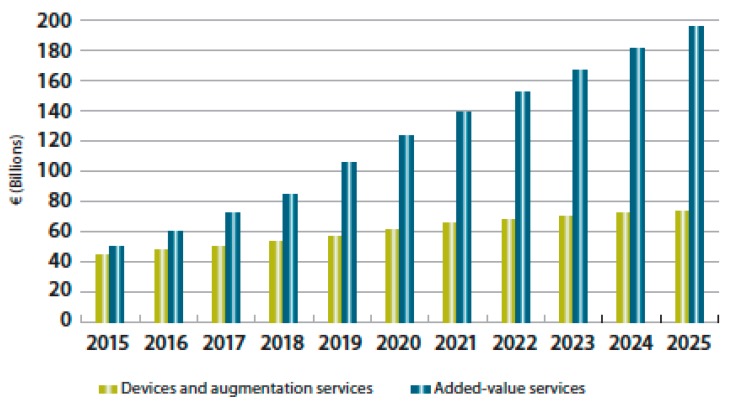
Revenue by type.

In this perspective, Reference [28] proposed a new conceptual framework for innovation and competitive advantage: The so-called service-dominant logic (SDL), as opposed to the traditional product-dominant logic (PDL). The relevance of this new paradigm and the urgency to embrace it cannot be neglected, since 70% of Western world gross domestic product derives from services [29], while the large majority of innovation research is focused on products or technology. This trend is confirmed in GNSS downstream markets by the progressive vertical integration (or extended offering) of product manufacturers and service providers, with a growing relevance of the latter [2]. In particular, “In 2015, the added-value [services] market size for the first time exceeded the combined size of GNSS devices and augmentation services. Their annual revenues will hit € 195 bln in 2025, which is more than 2.5 times higher than the expected GNSS device and service revenues that same year.” [2], as shown in Figure 4.

Since products become commodities almost as fast as they emerge, only services can provide a sustainable competitive advantage, as customers increasingly value experiences over products [27]. Reimagining a product as a service, as cloud-based software (and infrastructure- or platform-) as-a-service firms have done, transforms one-time software sales into ongoing service contracts. In this perspective, the proposed ICT cloud infrastructure can be a significant support to service providers, as it represents a technical innovation helpful in better meeting rapidly evolving user needs.

#### 3.2.4. Business Model Design and Risk Management

The growing development and spread of cloud-based services triggered investigation on the related business management, in particular, to shed new light on the most appropriate business model to be implemented and on effectively assess and mitigate risks. 

With an action design research study, reference [30] investigated viable business model options for PaaS. Both incumbent and nascent software providers need to cope with transforming, configuring, and calibrating their PaaS business model so to successfully face evolving customer expectations and competitive pressure. To this end, they proposed a methodology to identify and develop a suitable business model for cloud platforms.

In the case of a PaaS delivering GNSS-based solutions, a key parameter for the platform design is customer ownership, to be customized according to the trade-off between user requirements, the provider’s needs to avoid disclosure of industrial secrets, and the opportunity for the provider to exploit crowd-sourced information.

Concerning cloud-based service risks identification and management, reference [31] proposed four main mitigation actions, namely stakeholder engagement, technology development, innovation planning, and innovation control to address the most threatening risks, which can be related to the service itself, the technology adopted, and/or the processes undertaken for the delivery. This strategic approach can be helpful to draw a risk profile for a specific provider, and to set the most effective configuration of mitigation solutions accordingly. In the case of the proposed ICT infrastructure, the risks might widely vary according to the specific segment of the application, the kind and number of stakeholders involved and the technical components leveraged to deliver the GNSS high-accuracy integrated solution.

## 4. Discussion

This paper highlights the necessity of a new ICT infrastructure for innovative GNSS solutions based on user-driven technical requirements. As the novelty of the user-driven approach—both in technical feasibility and realization—the literature is poor for a comparison. The interesting and promising ICT architecture brings intermediate and final GNSS products, generated from TREASURE project innovative algorithms for the end users.

The computational functionalities and the evaluation of the performance of such a system are presented in Table 4. The evaluation is done through the technical questionnaire which each algorithm is executed individually on a minimum system requirement to approximate the performance. Even though the performance requirements are user-specific, this does not create difficulties for the validation process. The performance of each microservice is evaluated based on the execution time and the size of the output data derived from the technical questionnaire. 

Performance of the whole integrated system shall be assessed whenever implemented in terms of overall timing, introduced delays and jitters. Some benchmarks have been introduced by [10], in terms of timing performance according to different configurations (RAM, CPUs, etc.) of the virtual machines and different workloads. A similar approach can be adopted to assess our proposed system as a whole and each microservice whenever in place. However, each microservice is here individually evaluated in terms of the actual execution time in each local infrastructure through a technical questionnaire. The timing of each microservice will be assessed and expected to over-perform the minimum requirements reported in Table 4. 

Configuration of the infrastructure on the public cloud is predefined providing easy and cost-effective implementation than the private one. This is the case when the network design is an issue. While Ground-Based Scintillation Climatology (GBSC) software tool, being the GNSS application tested in [10], provides ionospheric products for end-users based on a private cloud infrastructure with a preliminary performance result, our approach is architecturally promoting a larger GNSS application which allocates different GNSS products and services for a wide range of customers in near real-time and post-processing methods. To prove the technical feasibility of such a system, a user-driven methodology is developed. The input and output data of each microservice is explained specifically. Our theoretical solution is coming with technical feasibility supported by actual users who need GNSS correction products for improving positioning algorithms and to the end-users who inquire positioning services targeted the GNSS mass market. 

From the market trends presented in the previous section, it can be understood how the proposed solution can better address evolving user needs on one hand, and support service providers’ success on the market due to the provision of GNSS solution through an improved and customizable infrastructure.

The ICT architecture developed, with the flexibility and comprehensiveness it offers, can effectively support GNSS service providers in deploying several complementary strategies, namely allowing for a reconciliation among industrialization (provision of standardized services) and customization, and the simultaneous offering of a core single service and a bundle of several services [32], thanks to flexibility and modularization. The possibility to simultaneously implement different market strategies increases the likelihood to engage a wider set of users, even active in different domains of applications, which might also become a valuable source of information for the future evolution of the architecture. 

The adoption of a multi-disciplinary perspective is meant to overcome the limits that are often faced by technology developed following a purely technical approach, detached from market trends and user needs. Any idea, regardless of how brilliant it is, needs to solve or improve an existing issue or intercept a latent one in order to be actually developed. Therefore, we intend to offer a contribution not only to the scientific community with the cloud-based ICT infrastructure, but also to propose an innovation that fits in the present economic and commercial environment. 

## 5. Conclusions

The present work binds innovative GNSS error mitigation algorithms to an optimized ICT system architecture for users of GNSS high-accuracy positioning. At the time of writing, the academic discourse on IaaS is still emerging, especially in the GNSS domain. The authors offer an original contribution to proposing a new ICT cloud infrastructure for GNSS-based services, in particular concerning the provision of positioning algorithms and error mitigations. The designed architecture boasts dynamic scalability, increased integrity, and greater agility of the ICT system. The novelty of the solution developed lies in a customized ICT architecture, obtained through unique and privileged access to user communities thanks to the H2020 project TREASURE, allowing the development of a solution entirely driven by user needs. The economic outlook of GNSS downstream markets evolution highlights the actual fit of the technology proposed in the real context, in particular, offering useful insights to service providers to gain a sustainable competitive advantage. The simultaneous adoption of the technical and commercial perspective is meant to offer interesting findings to both the scientific community and GNSS industry, creating synergies previously unexplored. As avenue for further research, it would be interesting to deploy a performance assessment of the algorithms and capabilities of the proposed ICT infrastructure. Such an exercise would allow for a more specific benchmark with the state-of-the-art performance, highlighting the actual novelty and advantages of the proposed solution. 

## Figures and Tables

**Table 1:** TREASURE innovative algorithms.

Algorithm ID	Algorithm Description
AID1	Ionospheric (total electron content (TEC)) corrections
AID2	Tropospheric corrections
AID3	Multi-channel signal processing
AID4	Ionospheric scintillation mitigation
AID5	Ionospheric scintillation corrections integration
AID6	Zenith tropospheric delay (ZTD) mitigation
AID7	Orbit and clock corrections

**Table 2:** Input data.

INPUT ID	Repository	Format	Size
IN1-R	Instituto Brasileiro de Geografia e Estatística (IBGE), Countering GNSS high-accuracy applications limitations due to ionospheric disturbances in Brazil (CALIBRA)	RTCM 3.X, RINEX, ASCII	35 MB
IN2-G-L	International GNSS Service (IGS), local network of the Netherlands and Australia	RINEX	5–10 MB
IN3-L	Remote/local personal computer (pc), storage units	IF sampled data (raw bits)	Depends on the RF front-end. 30 min~105 GB
IN4-G-R	Concept for ionospheric scintillation mitigation for professional GNSS in Latin America (CIGALA)/CALIBRA network, IGS, CHAIN, Northern Europe networks	Binary files for CIGALA/CALIBRA (SBF)	40 MB
IN5-G-R-L	International Earth Rotation and Reference Systems Service (IERS), University of Nottingham (UNOTT), NASA’s Navigation and Ancillary Information Facility (NAIF)/NASA Jet Propulsion Laboratory (JPL), IBGE, European Reference Frame (EUREF), National Geodetic Survey Continuously Operating Reference Station (NGS CORS), Crustal Dynamics Data Information System (CDDIS)/IGS	Proposed similar to IGS products (IONEX, RINEX)	100–200 MB
IN6-L	Local network/pc	RINEX, SSR. ASCII—Trimble, Leica proprietary binary manufacturer formats	106 GB
IN7-G	CDDIS/IGS, IERS	RINEX, SP3, RINEX CLK, ANTEX	Depends on the number of stations at each epoch

**Table 3:** Output data.

OUTPUT ID	Product Type	Format	Size
OUT1	Intermediate	IONEX/ASCII	5 MB
OUT2	Intermediate	RINEX	0.1–0.5 MB/site
OUT3	Intermediate/Final	MATLAB *.mat	Acquisition ~340 KB Tracking ~7 MB
OUT4	Intermediate	ASCII	100 KB
OUT5	Final	MATLAB *.mat	10.5 MB
OUT6	Intermediate/Final	SSR binary, ASCII	6 GB
OUT7	Final	RTCM SSR message	As current IGS products

**Table 4:** Computational functionalities and performance requirements.

Performance Requirement	Execution Time (per Site)	Size (per Day)
AID1	A few seconds	20 MB
AID2	One minute for Windows	1 MB
AID3	Not Required	Acquisition/satellite ~340 KB Tracking/satellite ~7 MB
AID4	Five hours	500 KB
AID5	50–120 seconds	10.5 MB
AID6	Variable	6 GB (41 stations in a week)
AID7	Real-time	In trade-off
